# Comparing Narcissism Measures in Their Confounding with Self-Esteem and Examining the Consequences for Their Relations with Personality

**DOI:** 10.3390/bs15111456

**Published:** 2025-10-26

**Authors:** Tobias Altmann, Marcus Roth

**Affiliations:** Department of Psychology, University of Duisburg-Essen, 45141 Essen, Germany

**Keywords:** narcissism, self-esteem, confoundment, assessment

## Abstract

Measures of narcissism often overlap with global self-esteem, risking that observed associations with outcomes may reflect associations of self-regard rather than actual narcissistic dispositions. The present study examined whether common narcissism instruments differ in their overlap with self-esteem and how this alters their associations with key personality domains. A sample of 337 participants completed multiple measures of narcissism, a global self-esteem measure as the control variable, and assessments of the Big Five, empathy, and aggression as personality correlates. Our results showed that overlap the measures of narcissism share with self-esteem varied considerably. Vulnerable scales showed the largest overlap and the greatest changes in correlations with the personality correlates after controlling for self-esteem. Grandiose and antagonistic measures were generally less affected, though noteworthy differences emerged between these instruments as well. We conclude that self-esteem overlap is a substantive but uneven measurement issue. Researchers cannot assume measures to be interchangeable. Our findings suggest that in order to isolate narcissistic dispositions from self-regard, researchers may need to select less affected instruments and/or report (additional) analyses controlling for self-esteem.

## 1. Introduction

Narcissistic personality tendencies have far-reaching consequences for social interaction and interpersonal functioning (Bettencourt et al., 2006). Individuals high in narcissistic traits are consistently characterized by low agreeableness, reduced empathy, and heightened interpersonal aggression, shaping relationship quality, conflict, and influence processes (Simard et al., 2023; Zajenkowski & Szymaniak, 2019). Yet efforts to understand narcissism’s role in personality and social behavior may be complicated by a measurement concern: many commonly used instruments appear to overlap with self-esteem (Rosenthal & Hooley, 2010). This potential confound has long been noted (Baumeister et al., 2000; Brummelman et al., 2016; Hyatt et al., 2018) but has not been systematically examined, leaving open the question of how strongly different measures are affected and to what extent such overlap distorts associations between narcissism and broader aspects of personality. The present study addresses this issue by directly comparing different narcissism measures, quantifying their degree of confounding with self-esteem, and examining how this influences their observed correlates with personality traits.

### 1.1. The Multidimensional Nature of Narcissism in Personality Research

Since the introduction of narcissistic personality disorder in the third edition of the Diagnostic and Statistical Manual of Mental Disorders (American Psychiatric Association, 1980), the concept of narcissism has not only gained prominence in clinical psychology but has also stimulated extensive research in personality psychology. In this context, narcissism is conceptualized as a personality trait that describes individual differences in non-clinical populations. Consistent with the broader tendency in personality research to assess traits through self-report, narcissism is measured almost exclusively via questionnaire instruments. One of the most prominent measures is the Narcissistic Personality Inventory (NPI; Raskin & Hall, 1979), which was developed based on DSM-III criteria and operationalizes narcissism as a primarily grandiose, unidimensional construct (Pincus & Lukowitsky, 2010). To this day, it remains the most widely used instrument for assessing narcissism (Foster & Raley, 2023).

In contrast to the early unidimensional conceptions, contemporary theory and measurement approaches increasingly conceptualize narcissism as a complex and multidimensional construct. The most prominent differentiation of narcissism is based on Wink (1991), who identified two dimensions underlying the narcissistic personality which he labeled grandiosity-exhibitionism (grandiose or overt narcissism) and vulnerability-sensitivity (vulnerable or covert narcissism). Grandiose narcissism involves an exhibitionistic tendency, the desire to maintain an overly positive self-image, and a strong need for admiration by others. Vulnerable narcissism, on the other hand, is characterized by a preoccupation with grandiose fantasies, vacillation between feelings of superiority and inferiority and a fragile sense of self. The admiration–rivalry distinction refers exclusively to the component of grandiosity and highlights that ostensibly grandiose expressions can be behaviorally and functionally heterogeneous (Back et al., 2013). Admiration reflects agentic self-promotion and social charm, whereas rivalry reflects antagonism and competitive hostility.

In addition to bi-dimensional models, there are other, more comprehensive attempts to describe narcissism with a broader perspective. The Five-Factor Narcissism approach positions narcissistic traits within the canonical Big Five taxonomy and maps a variety of narcissistic facets onto broader trait space, enabling a faceted view of agentic, antagonistic, and vulnerable features (Glover et al., 2012). More recent synthetic accounts propose a narcissism spectrum in which different expressions of narcissism occupy distinct loci along multiple dimensions (e.g., agentic versus antagonistic versus vulnerable) rather than forming a single continuum (Krizan & Herlache, 2018). Importantly, grandiose and vulnerable manifestations remain useful descriptive poles within this more complex topography.

### 1.2. Confounding with Self-Esteem

One of the central differences between clinical conceptions of narcissism and trait-based perspectives as mentioned above concerns its relation to self-esteem (Miller & Campbell, 2008). Classic clinical accounts (Kernberg, 1975; Kohut, 1971) emphasize narcissists’ low and fragile self-esteem, whereas trait perspectives often posit high self-esteem (Baumeister et al., 2000) and were unable to find robust support for the fragile self-esteem assumption of narcissism (Bosson et al., 2008). From a positive psychology perspective, this tension reflects a deeper distinction between narcissistic self-enhancement and forms of self-worth considered psychologically adaptive. Concepts such as authentic or optimal self-esteem (Kernis, 2003) describe a secure and noncontingent sense of self that supports well-being and resilience—qualities that stand in contrast to the defensive or inflated self-views often captured by narcissism measures. This broader perspective underscores why disentangling narcissism from self-esteem is not only a psychometric concern but also essential for differentiating maladaptive self-regard from genuinely healthy self-worth.

Rosenthal and Hooley (2010) argue that the discrepancies between perspectives do not reflect a fundamental theoretical divide but rather result from how narcissism is operationalized. Specifically, many items in narcissism questionnaires are confounded with self-esteem. According to Rosenthal and Hooley, this explains why narcissism as measured by the NPI often correlates unexpectedly with indicators of psychological health, such as lower depression and higher happiness (Rose, 2002; Sedikides et al., 2004).

To address this issue, Rosenthal and Hooley (2010) used three different approaches—expert ratings, item response theory, and exploratory factor analysis—to distinguish between NPI items that clearly captured narcissism and items that primarily reflected other constructs. They showed that only the weaker discriminating items were strongly associated with self-esteem and accounted for the NPI’s counterintuitive positive associations with psychological health, “suggesting that they measure self-esteem better than they measure narcissism” (Rosenthal & Hooley, 2010, p. 458). When these items were removed, the remaining NPI items were generally unrelated to self-esteem and psychological health but were positively related to aggression and anger. Thus, the self-esteem component embedded in the NPI appears to underlie its positive health correlates.

A subsequent meta-analysis of 54 studies confirmed these findings (Rosenthal et al., 2011). The results showed that the subset of NPI items failing to distinguish narcissists from non-narcissists was entirely responsible for the observed link between the NPI and psychological health. These items were strongly associated with self-esteem but not with aggression or anger.

However, the confounding with self-esteem in the measurement of narcissism is not limited to the NPI. As mentioned above, multidimensional views of narcissism currently prevail which have direct implications for its measurement and for the interpretation of empirical associations. Different instruments emphasize different portions of the narcissistic spectrum: some scales foreground agentic/admiration content (Back et al., 2013; M. Crowe et al., 2016; Raskin & Hall, 1979), whereas others emphasize vulnerability or antagonism (M. L. Crowe et al., 2018; Hendin & Cheek, 1997). Such variation matters because instruments that focus on agentic, admiring content tend to include items that resemble indicators of high self-esteem and assertive self-presentation; conversely, measures that emphasize vulnerability contain items that overlap with low self-esteem (Bosson et al., 2008; Rohmann et al., 2012). This heterogeneity in item content creates the potential for systematic differences in the degree to which measures are confounded with self-esteem, and thereby for differential distortion of associations with other personality constructs (Rosenthal & Hooley, 2010). It should be noted that narcissism and self-esteem are of course related but also conceptually and empirically distinct constructs (Brummelman et al., 2016; Campbell et al., 2002) which should be paralleled by their assessment.

From the multidimensional perspective the problem of self-esteem confounding generalizes in a theoretically tractable way: if a given instrument disproportionately samples the admiration/agentic pole of the spectrum, observed positive relations with markers of adaptive functioning (or attenuated relations with aggression and poor social functioning) may reflect captured variance in self-esteem rather than genuine narcissistic processes. Conversely, instruments emphasizing vulnerability or antagonism may yield stronger apparent links to neuroticism and aggression because they sample variance that overlaps with low self-esteem or negative affectivity.

These measurement-driven differences have concrete consequences for the literature on narcissism and personality. Prior work repeatedly reports that grandiose forms of narcissism are positively associated with extraversion and negatively with agreeableness, and that vulnerable forms relate strongly to neuroticism (Miller et al., 2011; Zajenkowski & Szymaniak, 2019); likewise, deficits in empathy and elevated aggression are among the more consistent correlates across many operationalizations (Baumeister et al., 2000; Kjaervik & Bushman, 2021; Simard et al., 2023; Urbonaviciute & Hepper, 2020). Yet if particular instruments differentially incorporate self-esteem content, then cross-study comparisons and theoretical inferences about the adaptive or maladaptive nature of specific narcissistic traits are rendered ambiguous. Some associations may reflect genuine links between narcissistic dispositions and personality, whereas others may be driven (largely or in part) by co-measured self-esteem. In order to compare and interpret findings based on different narcissism questionnaires, it is therefore essential to be able to evaluate the various questionnaires in terms of their overlap with self-esteem and the impact of this overlap on their associations with other constructs or outcome variables.

### 1.3. The Present Research

Against this background, the present study adopts an exploratory approach to examine how a set of commonly used measures of narcissism relate to key personality constructs (namely the Big Five, empathy, and aggression), and how those relationships change when variance associated with global self-esteem is statistically accounted for. This approach addresses two intertwined questions: First, to what extent do different measures of narcissism capture variance attributable to self-esteem? To address this question, we employ various questionnaires to differentiate various facets of narcissism. This allows us to identify which components of narcissism, and which instruments, show the strongest confounding with self-esteem. We specifically expected the following results. Hypothesis 1: Measures of grandiose narcissism will be positively associated with self-esteem (due to their inclusion of extraversion-related content). Hypothesis 2: Measures of vulnerable narcissism will be negatively associated with self-esteem (given their emphasis on neuroticism-related content; Miller et al., 2017).

Second, to what extent do observed correlations between narcissism and other constructs depend on variance shared with self-esteem? Beyond health-related outcomes, we examine whether self-esteem accounts for the associations between narcissism and other personality traits within a broader trait network. By contrasting measures that emphasize admiration/agentic, antagonistic/rivalrous, and vulnerable aspects of narcissism, this study also helps clarify which associations remain robust when controlling for self-esteem and which may instead reflect measurement artifacts due to self-esteem confounding. Due to this exploratory approach, we did not phrase a specific hypothesis regarding this second question.

## 2. Method

### 2.1. Sample and Procedure

Power analysis with GPower version 3.1 (Faul et al., 2009) suggested a minimally required sample size of 138 to detect correlations of 0.30 with a power of 0.95. However, (Schönbrodt & Perugini, 2013) showed that correlation coefficients tend to stabilize in samples of around 250 participants which we aimed for as a minimum in the present study.

Participants were recruited at a German university via leaflets and social media posts. Assessments were conducted online (Altmann & Kapoor, 2021). Participants were excluded if they provided incomplete data. The final sample contained 337 participants of which 63.8% identified as women, 35.6% as men, and 0.6% as nonbinary. The mean age was 21.0 years (*SD* = 3.5).

### 2.2. Measures

All measures were administered in German. If not described differently, a five-point rating scale from 1 “does not apply to me” to 5 “applies to me” was used.

We selected narcissism inventories that are most commonly used in the current literature. Consequently, some facets of narcissism (e.g., grandiose and vulnerable narcissism) are assessed by multiple inventories, additionally allowing readers to determine whether the observed effects are dependent on the specific measure or reflect the underlying construct itself. We used the following inventories as measures of narcissism: A short version of the Narcissistic Personality Inventory with 15-items (NPI; Raskin & Hall, 1979; Schütz et al., 2004) was used which measures grandiose narcissism. Every item contains two options of which the participants have to choose the one that describes them best (e.g., Option 1 “When people compliment me, I sometimes get embarrassed” or Option 2 “I know that I am good because everybody keeps telling me so.”). A second measure of grandiose narcissism we applied was the Narcissistic Grandiosity Scale (NGS; Rosenthal et al., 2020). It contains 16 adjectives, and participants rate how well each adjective describes them. The 30-item version of the Five-Factor Narcissism Inventory was used (FFNI; Glover et al., 2012; Jauk et al., 2023) as a multidimensional inventory. It offers the three subscales agentic narcissism, antagonism, and neurotic narcissism as well as the two subscales grandiose narcissism and vulnerable narcissism. As a measure of vulnerable narcissism, we used the Hypersensitive Narcissism Scale (HSNS; Hendin & Cheek, 1997) which contains ten items. Another measure of vulnerable narcissism we applied was the Narcissistic Vulnerability Scale (NVS; M. L. Crowe et al., 2018). It contains 12 items, and participants rate how well each adjective describes them. Lastly, we used the Narcissistic Admiration and Rivalry Questionnaire (NARQ; Back et al., 2013). It contains the two subscales admiration and rivalry with nine items each.

To assess self-esteem, we used the established ten-item Rosenberg Self-Esteem Scale (RSES; Rosenberg, 1965; von Collani & Herzberg, 2003).

We assessed the following additional aspects of personality with the following inventories: The Big Five were assessed using the SOEP version of the Big Five Inventory (BFI; Schupp & Gerlitz, 2008). Its fifteen items assess the five dimensions agreeableness, conscientiousness, extraversion, neuroticism, and openness with three items each. We note that internal consistency scores for agreeableness and openness were critically low (see Table 1), likely due to the small number of items representing these broad constructs. Nevertheless, we included the corresponding results, as low alpha coefficients are generally not considered problematic in group-level analyses (Gosling et al., 2003; Ziegler et al., 2014), unlike in individual assessments. Moreover, the inventory demonstrated acceptable test–retest reliability and validity (Hahn et al., 2012), supporting its suitability for the present study.

The Toronto Empathy Questionnaire (TEQ; Janelt et al., 2024; Spreng et al., 2009) was used to assess empathy. Lastly, the 12-item version of the Buss-Perry Aggression Questionnaire (AQ; Bryant & Smith, 2001; Buss & Perry, 1992; Werner & von Collani, 2004) was used to assess the disposition to react with aggression, anger, and hostility in verbal or physical forms.

## 3. Results

Table 1 displays the descriptive statistics of all variables in the study. Cronbach’s alphas (comparable values were found using McDonald’s omega) were acceptable except for agreeableness and openness of the Big Five. Although this index has been argued to be of lesser importance in sample statistics (Gosling et al., 2003; Tavakol & Dennick, 2011) compared, for instance, to its relevance in the assessment of individuals, the respective results will have to be treated with caution.

The (uncontrolled) bivariate correlations between the various measures of narcissism and the further aspects of personality are presented in Table 2. The penultimate column shows their correlations with self-esteem which ranges between a nonsignificant value of −0.04 with grandiose narcissism as measured by the Five-Factor Narcissism Inventory, −0.56 with vulnerable narcissism of the same inventory on the negative side, and 0.35 with the admiration subscale of the Narcissistic Admiration and Rivalry Questionnaire on the positive side. The last column presents the proportion of covariance R^2^ ranging between 0.2% with the grandiose narcissism subscale and 30.9% with the vulnerable narcissism subscale from the Five-Factor Narcissism Inventory.

Table 3 contains the respective partial correlations when controlling for self-esteem. To provide a quick and intuitive overview of the differences that occurred when comparing the uncontrolled correlations (Table 2) to the partial correlations controlling for self-esteem (Table 3), we calculated their absolute differences which are presented in Table 4. These differences were then tested for significance using a bootstrap procedure with 2000 bootstrapped samples to account for their nonindependence (Davison & Hinkley, 2013; Meng et al., 1992). As can be seen in Table 4, most differences were found to be significant and ranged between −0.23 (between vulnerable narcissism measured by the Five-Factor Narcissism Inventory and extraversion), and 0.28 (between neurotic narcissism measured by the Five-Factor Narcissism Inventory and aggression).

In Table 4, we also implemented a temperature-scale-based heatmap as supportive visualization based on the absolute values of the differences between uncontrolled and controlled correlations. White fields indicate smaller differences; red fields indicate larger differences. Thus, for the measures of narcissism with mostly red fields, the issue of confounding with self-esteem is more influential compared to the measures of narcissism with mostly white fields.

Lastly, in an attempt to ease interpretation and applicability of the present findings, we computed the rank of each of the measures of narcissism regarding how large a difference occurred between their uncontrolled and controlled correlations within each dependent variable (i.e., Big Five, empathy, and aggression). This was done across the measures of narcissism for each of the dependent variables separately (i.e., per column). We then calculated the average rank for each measure of narcissism across the dependent variables (i.e., per line). This average score is presented in the last column of Table 4 and represents the order of measures of narcissism in how strongly their confounding with self-esteem affects their correlational associations with other constructs (i.e., Big Five, empathy, and aggression).

The top four ranks (largest impact of confounded self-esteem) were occupied by neurotic narcissism and vulnerable narcissism as measured by the Five-Factor Narcissism Inventory, vulnerable narcissism as measured by the Hypersensitive Narcissism Scale, and vulnerable narcissism as measured by Narcissistic Vulnerability Scale. The bottom three ranks were occupied by grandiose narcissism, agentic narcissism, and antagonism all measured by the Five-Factor Narcissism Inventory. These ranks are of course closely related (yet not identical) to the last column of Table 2 which contains the proportions of variance shared between each measure of narcissism and self-esteem.

## 4. Discussion

This study examined the extent to which commonly used measures of narcissism overlap with global self-esteem and how that overlap alters observed associations between narcissism and broad personality characteristics. Although the potential for self-esteem to confound narcissism assessment has been raised previously (Rosenthal & Hooley, 2010), it has not been systematically compared across the wide range of instruments now in use. By directly juxtaposing multiple grandiose, vulnerable, and facet-level inventories in a single sample and by comparing zero-order correlations with partial correlations that control for self-esteem, we were able to quantify how strongly self-esteem is embedded in different measures and how much this embedding affects conclusions about narcissism’s correlates.

### 4.1. Summary and Interpretation of Key Findings

The degree of overlap with self-esteem varied markedly across instruments. The most salient and consistent result was that measures of neurotic and vulnerable narcissism showed the largest overlap with self-esteem and the greatest changes in their pattern of personality correlations after controlling for self-esteem. Specifically, the Five-Factor Narcissism Model (Glover et al., 2012) neurotic and vulnerable narcissism subscales, the Hypersensitive Narcissism Scale (Hendin & Cheek, 1997), and the Narcissistic Vulnerability Scale (M. L. Crowe et al., 2018) occupied the top four ranks in the order of how strongly their confounding with self-esteem affects their correlational associations with other constructs. Consistently, they also showed the largest shared variance with self-esteem (ranging between around 24 to 31%). For instance, the FFNI vulnerable narcissism subscale’s bivariate correlation with neuroticism fell from 0.42 to 0.21 after controlling for self-esteem, and its correlation with aggression decreased from 0.45 to 0.30. These changes indicate that a substantial portion of the apparent associations between vulnerable narcissism scales and several personality traits may reflect shared variance with self-esteem.

By contrast, measures of grandiose narcissism were generally less affected, but nevertheless important differences emerged among them. The Narcissistic Grandiosity Scale (M. Crowe et al., 2016), for example, showed moderate confounding and was ranked comparable to the Narcissistic Personality Inventory (Raskin & Hall, 1979), whereas the Five-Factor Narcissism Model (Glover et al., 2012) grandiose narcissism subscale was among the least affected of all instruments. This heterogeneity demonstrates that even within the grandiose domain, instruments are not interchangeable.

It seems worth noticing that among all instruments, the FFNI showed the broadest internal range in its correlations with self-esteem, from close to zero for its grandiose narcissism subscale to strong negative associations for its vulnerable and neurotic subscales. Thus, the grandiose factor appears relatively uncontaminated in the sense that it captures self-enhancing tendencies largely independent of global self-regard, whereas the vulnerable and neurotic factors represent defensive and self-critical aspects tightly linked to low self-esteem. This pattern should be considered in light of how self-esteem was assessed. The Rosenberg Self-Esteem Scale primarily captures a continuum from maladaptive low self-worth to adaptive high self-esteem, without differentiating between adaptive high or optimal and exaggeratedly inflated forms of self-regard. Consequently, any potential association between grandiose narcissism and overly enhanced self-esteem may be underestimated due to this ceiling effect, whereas links between vulnerable narcissism and maladaptively low self-esteem are more readily detected.

The present findings extend and refine prior critiques that focused primarily on individual instruments (Rosenthal & Hooley, 2010) by demonstrating that the self-esteem overlap is a broader, but heterogenous and therefore measurement-specific phenomenon. Importantly, our results show that the phenomenon is not limited to agentic grandiosity measures: vulnerable scales—despite their conceptual emphasis on insecurity and negative self-views—were particularly affected.

### 4.2. Practical Implications

The principal practical implication of our findings may be that the choice of narcissism measure matters (as has been argued regarding other constructs such as sensation seeking and empathy as well; Altmann et al., 2019; Stosic et al., 2022). This consideration is particularly important in applied settings such as clinical assessment or organizational psychology, where conclusions about narcissistic traits can inform diagnostic impressions or personnel decisions, as well as in research contexts where narcissism may be examined in relation to personality, well-being, or interpersonal functioning. Practitioners and researchers who intend to assess narcissism per se (i.e., the personality disposition distinct from global self-esteem) cannot necessarily assume that different instruments will produce equivalent results. In particular, if the aim is to target narcissistic features that are relatively independent of self-esteem (e.g., antagonism, facets of grandiosity), measures should be preferred that showed low shared variance with self-esteem in our comparison (for example, the FFNI grandiose narcissism and antagonism subscales). If a study uses measures that showed substantial overlap with self-esteem (notably the vulnerable measures examined here), authors may want to consider explicitly addressing this overlap, either by including global self-esteem as a covariate and reporting both uncontrolled and controlled results, or by justifying theoretically why self-esteem shared variance is acceptable for the research question at hand. Reporting both uncontrolled and controlled results is advisable because partialling can sometimes remove variance that is substantively relevant when self-esteem is part of the phenomenon under study (Lynam et al., 2006).

Thus, this issue is not merely statistical but fundamentally tied to the theoretical conceptualization of narcissism. Whether self-esteem should be treated as part of the narcissism construct or as shared but nondefining variance depends on the theoretical framework applied. From clinical perspectives, particularly those following Kernberg (1975) and Kohut (1971), fluctuations in self-esteem are central to narcissistic pathology: fragile or chronically low self-esteem is viewed as a defining feature of narcissism and, in more severe forms, as a consequence of impaired self-cohesion. Under such views, statistically removing self-esteem may inadvertently remove variance essential to the construct itself. In contrast, personality psychological approaches often (though not homogenously) conceptualize self-esteem as a related but distinct disposition (Bosson et al., 2008; Brummelman et al., 2016; Campbell et al., 2002). From this standpoint, controlling for self-esteem helps isolate the uniquely narcissistic aspects of self-regard from general self-evaluation. Recognizing this theoretical tension underscores that the decision to control for self-esteem is not purely statistical but should be guided by the conceptualization of narcissism that informs a given study.

### 4.3. Limitations

Several limitations of the present study should be mentioned. First, the sample is a young and educated sample which limits the generalizability of the present findings. Patterns of measurement overlap could differ in older, clinical, non-western, or more heterogeneous community samples. For instance, age-related differences in self-esteem stability (Meier et al., 2011) could influence how strongly narcissism measures overlap with self-esteem, potentially leading to weaker confounding in older samples. Similarly, clinical populations may show greater variability in self-esteem regulation and defensive functioning, which could alter the extent or direction of overlap between narcissism and self-esteem measures.

Second, internal consistency for agreeableness and openness was relatively low (see Table 1). Low reliability, particularly Cronbach’s alpha values around 0.50, can attenuate observed correlations (classical test theory), potentially leading to underestimation of the true associations between these personality domains and narcissism measures. As our distortion indices are based on correlations, reduced reliability may have dampened these indices, thereby possibly underrepresenting the extent to which overlap with self-esteem alters associations with agreeableness and openness. Nevertheless, these scales were retained because low alpha coefficients are generally less problematic in group-level analyses (Gosling et al., 2003; Ziegler et al., 2014), and the inventory has demonstrated adequate test–retest reliability and validity (Hahn et al., 2012). Despite this, the low internal consistency suggests that the results involving agreeableness and openness should be interpreted with caution, as the observed effects may underestimate the true relationships. Future research could benefit from using more comprehensive assessments of these traits to ensure more reliable estimates and a clearer understanding of their associations with narcissism and self-esteem overlap.

Third, there is an ongoing conceptual debate about whether certain aspects of narcissism legitimately include self-evaluative content (Bosson et al., 2008; Brummelman et al., 2016; Campbell et al., 2002). Statistically removing self-esteem may therefore remove variance that is theoretically meaningful for some research questions (Lynam et al., 2006), as elaborated above.

### 4.4. Future Directions

The present results open multiple directions for advancing the understanding of how narcissism and self-esteem are intertwined. A developmental perspective may clarify how narcissism and self-esteem co-develop over time, and whether grandiose and vulnerable forms follow distinct trajectories. Longitudinal studies could test whether fragile self-esteem precedes increases in vulnerable narcissism or whether stable self-esteem buffers against maladaptive expressions of grandiosity (Bosson et al., 2008). Beyond trait levels, dynamic designs such as experience-sampling or laboratory paradigms could examine how momentary changes in self-esteem interact with narcissistic tendencies following success, rejection, or social evaluation (Geukes et al., 2017). Finally, cross-cultural and contextual approaches may reveal whether the overlap between narcissism and self-esteem varies across sociocultural settings that differ in norms surrounding self-enhancement, modesty, or individualism (Liu & Zheng, 2025). Such work would help determine to what extent the confounding observed here reflects universal psychological mechanisms or context-dependent expressions of self-regard.

## 5. Conclusions

The present study shows that self-esteem overlap is a substantive but uneven measurement issue in narcissism research. It was especially pronounced for vulnerable narcissism measures, which displayed the strongest confounding with self-esteem. Grandiose measures were generally less affected, yet still differed in their degree of overlap, indicating that even within this domain instruments cannot be deemed interchangeable.

As practical implications, researchers may want to consider their choice of measure and explicitly account for the role of self-esteem when interpreting associations of narcissism with personality and other outcomes. Preregistering and reporting both uncontrolled and self-esteem-controlled results can reduce the risk of distorted conclusions and promote more precise assessment of narcissism in personality research.

## Figures and Tables

**Table 1:** Descriptive Statistics of the Study Variables.

	Cronbach’s Alpha	*M*	*SD*	Min	Max
Narcissism					
Grandiose Narcissism (NPI)	0.77	0.29	0.21	0.0	0.9
Grandiose Narcissism (NGS)	0.93	1.99	0.76	1.0	4.8
Grandiose Narcissism (FFNI)	0.76	2.39	0.55	1.3	4.2
Vulnerable Narcissism (FFNI)	0.62	3.09	0.71	1.1	4.9
Vulnerable Narcissism (HSNS)	0.68	2.90	0.60	1.2	4.6
Vulnerable Narcissism (NVS)	0.87	2.33	0.79	1.0	4.4
Agentic Narcissism (FFNI)	0.75	2.59	0.81	1.0	4.9
Antagonism (FFNI)	0.74	2.29	0.57	1.0	4.3
Neurotic Narcissism (FFNI)	0.80	3.33	0.91	1.0	5.0
Admiration (NARQ)	0.83	2.73	0.71	1.0	4.8
Rivalry (NARQ)	0.82	2.10	0.70	1.0	4.8
Self-esteem					
Self-esteem (RSES)	0.87	3.42	0.80	1.1	5.0
Further personality					
Agreeableness (BFI)	0.43	3.84	0.69	2.0	5.0
Conscientiousness (BFI)	0.70	3.47	0.85	1.0	5.0
Extraversion (BFI)	0.82	3.22	1.01	1.0	5.0
Neuroticism (BFI)	0.70	3.60	0.89	1.3	5.0
Openness (BFI)	0.56	3.62	0.81	1.3	5.0
Empathy (TEQ)	0.80	3.98	0.52	2.4	5.0
Aggression (AQ)	0.82	2.24	0.65	1.0	4.3

Note. NPI = Narcissistic Personality Inventory. NGS = Narcissistic Grandiosity Scale. FFNI = Five-Factor Narcissism Inventory. HSNS = Hypersensitive Narcissism Scale. NVS = Narcissistic Vulnerability Scale. NARQ = Narcissistic Admiration and Rivalry Questionnaire. RSES = Rosenberg Self-Esteem Scale. BFI = Big Five Inventory. TEQ = Toronto Empathy Questionnaire. AQ = Buss-Perry Aggression Questionnaire.

**Table 2:** Bivariate Correlations Between the Measures of Narcissism and Personality.

	Agreeableness	Conscientiousness	Extraversion	Neuroticism	Openness	Empathy	Aggression	Self-Esteem	*R*^2^ Self-Esteem
Grandiose Narcissism (NPI)	−0.27 ***	0.02	0.40 ***	−0.38 ***	0.11 *	−0.25 ***	0.23 ***	0.24 ***	5.6%
Grandiose Narcissism (NGS)	−0.27 ***	0.02	0.19 ***	−0.33 ***	0.04	−0.32 ***	0.26 ***	0.23 ***	5.2%
Grandiose Narcissism (FFNI)	−0.40 ***	−0.17 **	0.11	−0.29 ***	0.00	−0.43 ***	0.51 ***	−0.04	0.2%
Vulnerable Narcissism (FFNI)	−0.14 *	−0.18 **	−0.29 ***	0.42 ***	−0.02	0.04	0.45 ***	−0.56 ***	30.9%
Vulnerable Narcissism (HSNS)	−0.17 **	−0.10	−0.41 ***	0.38 ***	0.06	−0.12 *	0.39 ***	−0.49 ***	23.8%
Vulnerable Narcissism (NVS)	−0.33 ***	−0.22 ***	−0.26 ***	0.25 ***	−0.06	−0.14 *	0.58 ***	−0.53 ***	28.2%
Agentic Narcissism (FFNI)	−0.29 ***	−0.05	0.22 ***	−0.26 ***	0.13 *	−0.26 ***	0.35 ***	0.11 *	1.2%
Antagonism (FFNI)	−0.45 ***	−0.25 ***	−0.02	−0.25 ***	−0.12 *	−0.51 ***	0.63 ***	−0.15 **	2.3%
Neurotic Narcissism (FFNI)	0.05	−0.10	−0.29 ***	0.53 ***	0.02	0.24 ***	0.12 *	−0.55 ***	30.1%
Admiration (NARQ)	−0.17 **	0.04	0.35 ***	−0.32 ***	0.18 **	−0.26 ***	0.18 ***	0.35 ***	12.4%
Rivalry (NARQ)	−0.55 ***	−0.29 ***	−0.14 **	−0.06	−0.17 **	−0.52 ***	0.60 ***	−0.25 ***	6.2%

Note. Abbreviations of measures are explained in the Method section and Table 1. * *p* < 0.05. ** *p* < 0.01. *** *p* < 0.001.

**Table 3:** Partial Correlations Between the Measures of Narcissism and Personality Controlling for Self-Esteem.

	Agreeableness	Conscientiousness	Extraversion	Neuroticism	Openness	Empathy	Aggression
Grandiose Narcissism (NPI)	−0.35 ***	−0.06	0.34 ***	−0.32 ***	0.07	−0.28 ***	0.38 ***
Grandiose Narcissism (NGS)	−0.34 ***	−0.05	0.10	−0.26 ***	0.00	−0.35 ***	0.41 ***
Grandiose Narcissism (FFNI)	−0.40 ***	−0.17 **	0.14 *	−0.34 ***	0.00	−0.43 ***	0.54 ***
Vulnerable Narcissism (FFNI)	0.00	0.00	−0.06	0.23 ***	0.09	0.12 *	0.27 ***
Vulnerable Narcissism (HSNS)	−0.06	0.07	−0.25 ***	0.20 ***	0.17 **	−0.08	0.23 ***
Vulnerable Narcissism (NVS)	−0.25 ***	−0.07	−0.04	0.01	0.04	−0.10	0.46 ***
Agentic Narcissism (FFNI)	−0.32 ***	−0.08	0.19 ***	−0.24 ***	0.11 *	−0.27 ***	0.45 ***
Antagonism (FFNI)	−0.43 ***	−0.21 ***	0.05	−0.36 ***	−0.10	−0.50 ***	0.63 ***
Neurotic Narcissism (FFNI)	0.23 ***	0.09	−0.07	0.38 ***	0.14 *	0.36 ***	−0.17 **
Admiration (NARQ)	−0.28 ***	−0.08	0.23 ***	−0.19 ***	0.13 *	−0.32 ***	0.40 ***
Rivalry (NARQ)	−0.52 ***	−0.23 ***	−0.04	−0.20 ***	−0.13 *	−0.51 ***	0.56 ***

Note. Abbreviations of measures are explained in the Method section and Table 1. * *p* < 0.05. ** *p* < 0.01. *** *p* < 0.001.

**Table 4:** Heatmap of Absolute Differences Between Uncontrolled and Partial Correlations with Bootstrapped 95% Confidence Intervals.

	Agreeableness	Conscientiousness	Extraversion	Neuroticism	Openness	Empathy	Aggression	Mean of Ranks
Grandiose Narcissism (NPI)	0.08 [0.04, 0.12] *	0.08 [0.04, 0.12] *	0.06 [0.02, 0.10] *	−0.06 [−0.11, −0.02] *	0.04 [0.01, 0.07] *	0.03 [0.01, 0.07] *	−0.15 [−0.21, −0.10] *	6.7
Grandiose Narcissism (NGS)	0.07 [0.04, 0.11] *	0.08 [0.04, 0.12] *	0.09 [0.04, 0.14] *	−0.07 [−0.12, −0.02] *	0.04 [0.01, 0.07] *	0.04 [0.01, 0.07] *	−0.15 [−0.21, −0.10] *	6.1
Grandiose Narcissism (FFNI)	0.00 [−0.02, 0.03]	−0.00 [−0.04, 0.03]	−0.03 [−0.08, 0.01]	0.06 [0.01, 0.10] *	−0.01 [−0.03, 0.01]	−0.00 [−0.01, 0.01]	−0.04 [−0.07, 0.00]	10.3
Vulnerable Narcissism (FFNI)	−0.13 [−0.19, −0.07] *	−0.17 [−0.24, −0.11] *	−0.23 [−0.30, −0.16] *	0.19 [0.13, 0.25] *	−0.11 [−0.19, −0.05] *	−0.08 [−0.14, −0.02] *	0.17 [0.12, 0.23] *	2.0
Vulnerable Narcissism (HSNS)	−0.11 [−0.17, −0.05] *	−0.16 [−0.23, −0.11] *	−0.16 [−0.22, −0.10] *	0.17 [0.12, 0.23] *	−0.11 [−0.17, −0.05] *	−0.04 [−0.10, 0.01]	0.16 [0.11, 0.22] *	3.3
Vulnerable Narcissism (NVS)	−0.08 [−0.14, −0.03] *	−0.15 [−0.22, −0.09] *	−0.22 [−0.30, −0.15] *	0.23 [0.17, 0.30] *	−0.10 [−0.16, −0.04] *	−0.04 [−0.10, 0.02]	0.12 [0.08, 0.18] *	3.9
Agentic Narcissism (FFNI)	0.04 [0.01, 0.07] *	0.04 [0.00, 0.08]	0.03 [−0.02, 0.07]	−0.02 [−0.07, 0.02]	0.02 [−0.00, 0.04]	0.01 [0.00, 0.04]	−0.09 [−0.14, −0.05] *	9.0
Antagonism (FFNI)	−0.02 [−0.04, 0.00]	−0.03 [−0.07, −0.00]	−0.07 [−0.12, −0.03] *	0.11 [0.06, 0.16] *	−0.02 [−0.05, −0.00]	−0.01 [−0.02, 0.00]	−0.00 [−0.03, 0.03]	9.0
Neurotic Narcissism (FFNI)	−0.17 [−0.24, −0.11] *	−0.19 [−0.26, −0.13] *	−0.22 [−0.29, −0.16] *	0.15 [0.10, 0.20] *	−0.12 [−0.19, −0.06] *	−0.12 [−0.19, −0.06] *	0.28 [0.22, 0.35] *	1.6
Admiration (NARQ)	0.11 [0.06, 0.16] *	0.12 [0.07, 0.17] *	0.12 [0.07, 0.18] *	−0.13 [−0.18, −0.08] *	0.05 [0.01, 0.10] *	0.06 [0.02, 0.10] *	−0.22 [−0.29, −0.15] *	4.1
Rivalry (NARQ)	−0.03 [−0.05, −0.01] *	−0.06 [−0.11, −0.02] *	−0.10 [−0.16, −0.05] *	0.14 [0.08, 0.20] *	−0.04 [−0.07, −0.01] *	−0.01 [−0.03, 0.01]	0.04 [0.00, 0.07]	7.3

Note. Heat colors range between white for scores close to zero and dark red for scores further from zero. Asterisks (*) denote bootstrapped confidence intervals which do not include zero. Abbreviations of measures are explained in the Method section and Table 1.

## Data Availability

The data presented in the study are being made openly available in the Open Science Framework at https://osf.io/ma3fg.
